# Renal venous sampling assisted the diagnosis of juxtaglomerular cell tumor: a case report and literature review

**DOI:** 10.3389/fonc.2023.1298684

**Published:** 2024-01-18

**Authors:** Di-en Yan, Hong-bing He, Jian-ping Guo, Yu-lan Wang, Dan-ping Peng, Huan-huan Zheng, Xiao-zi Zhou, Jin-xiang Fu, Mei-li Wang, Xian Luo, Yun-feng Shen

**Affiliations:** ^1^ Department of Endocrinology, Ji’an Central Hospital, Ji’an, Jiangxi, China; ^2^ Department of Endocrinology and Metabolism, The Second Affiliated Hospital of Nanchang University, Nanchang, Jiangxi, China

**Keywords:** renal venous sampling, renin, juxtaglomerular cell tumor, resistant hypertension, hypokalemia

## Abstract

Juxtaglomerular cell tumor (JCT) is an endocrine tumor marked by elevated renin levels and high blood pressure. This case report presents the clinical findings of a 47-year-old woman with a history of recurrent hypokalemia, headaches, hypertension, and increased plasma renin activity (PRA). Dynamic enhanced magnetic resonance imaging (MRI) revealed a small nodule on the upper part of the right kidney. Selective renal venous sampling indicated a higher PRA only in the right upper pole renal vein. The patient underwent surgical removal of the right kidney mass, and the pathology results confirmed the diagnosis of JCT. This case underscores the importance of conducting selective renal venous sampling for accurate JCT diagnosis.

## Introduction

Juxtaglomerular cell tumors (JCTs) secrete the enzyme renin, leading to overstimulation of the renin–angiotensin–aldosterone system. JCT is a rare condition first described by Robertson et al. in 1967 (1), causing severe hypertension and hypokalemia. JCT has been documented in fewer than 200 cases and predominantly affects women (2:1) (2). The prevalence of JCT has been increasing, especially with advances in renal venous sampling, which aids in detecting small tumors. In this report, we present a case of JCT in a patient with nearly 20 years of resistant hypertension, diagnosed using selective renal venous sampling.

## Case description

A 47-year-old woman with a family history of high blood pressure had been struggling with persistent resistant hypertension for nearly two decades without any diagnostic tests or treatment on September 20, 2021. Upon admission, her blood pressure was measured at 180/100 mmHg despite taking nifedipine (30 mg/day), irbesartan (150 mg/day), and atenolol (25 mg/day). She also experienced headaches and felt weak. A physical examination revealed no apparent abnormalities. Her potassium level was 2.8 mmol/L (within the normal range of 3.5–5.5 mmol/L), and all other standard biomarkers were within normal limits. Biochemical measurements of plasma renin activity (PRA), plasma aldosterone concentration (PAC), and angiotensin II were carried out with automated chemiluminescence immunoassays (Sinbe, Shenzhen, China). The endocrine tests indicated that PRA was 13.6 (normal range: 0.15–2.33 ng/mL/h), with repeat PRA measurements ranging from 10 to 27 ng/mL/h. Her PAC was 280 (within the normal range of 12–310 pg/mL), and her angiotensin II level was 210 (normal range: 25–245 pg/mL). Using 24-h ambulatory blood pressure monitoring, the results showed an average systolic and diastolic blood pressure of 184/117 mmHg. Notably, the non-contrast CT and MRI scans of the abdomen appeared normal, and Doppler ultrasonography ruled out renal artery stenosis. The patient was diagnosed with resistant hypertension, elevated PRA, and hypokalemia and was prescribed potassium chloride (KCl) supplementation (1.0 g, three times a day). Other endocrine tests, including dehydroepiandrosterone-sulfate, adrenocorticotropin, cortisol, thyroid-stimulating hormone, free triiodothyronine, free thyroxin, and catecholamines, all fell within normal ranges. Continuous infusion of urapidil maintained her blood pressure at 120–140/80–100 mmHg.

**Figure 1:**
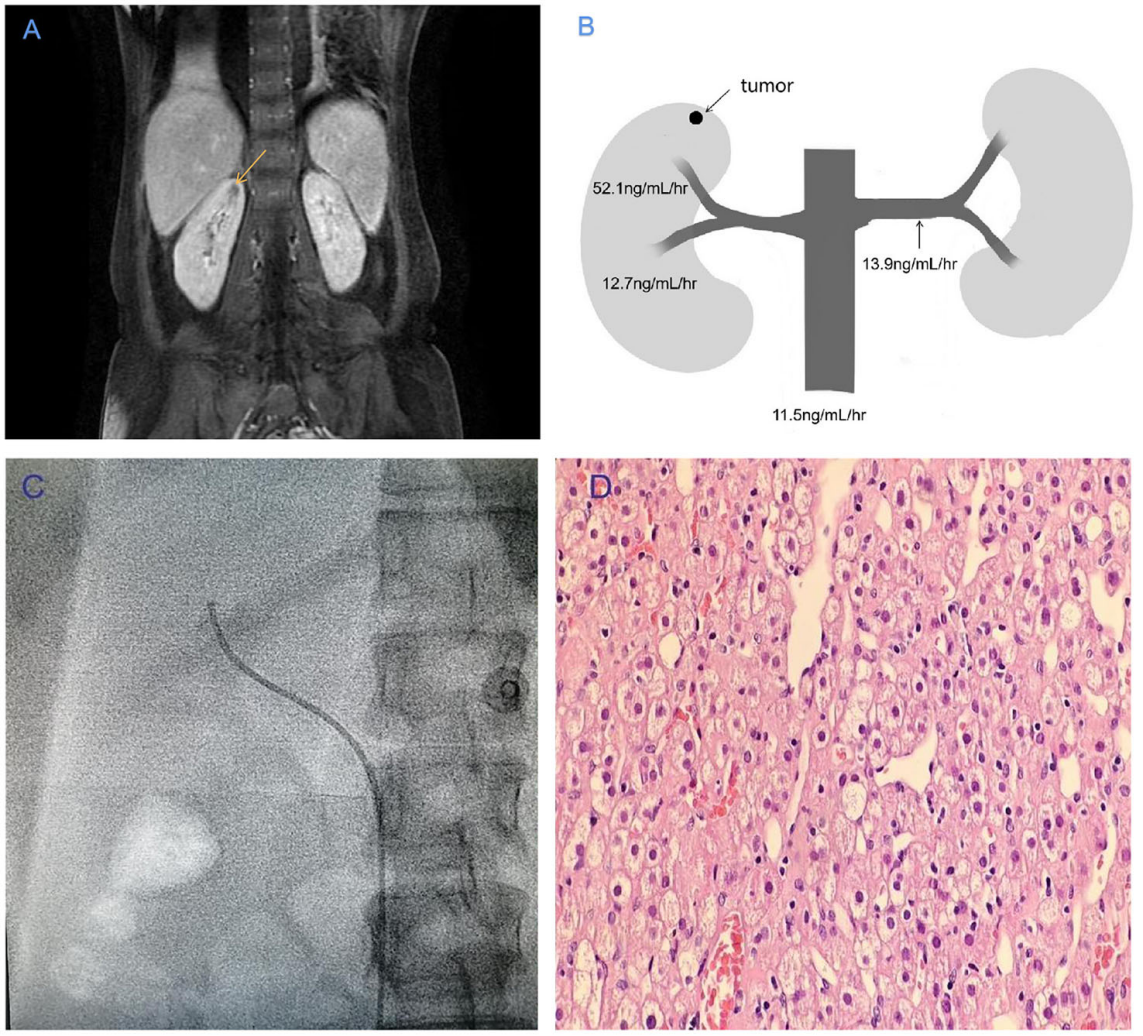
**(A)** T2-weighted MRI showing a tumor with a high signal intensity (indicated by the yellow arrow) in the right superior pole of the renal vein. **(B)** Plasma renin activity values at various sampling points in the vasculature of the renal veins. **(C)** Upper branch of the right renal vein in angiography. **(D)** The tumor morphology was observed using hematoxylin–eosin staining under a light microscope (10 × 40).

The non-enhanced CT scan of the abdomen appeared normal, but dynamic enhanced MRI revealed a small, irregular nodule measuring 0.6 cm in diameter on the upper part of the right kidney in the T2-weighted MRI, as shown in **Figure 1A**. The primary features of this case included resistant hypertension, elevated PRA, and hypokalemia. Pheochromocytoma, aortitis, and malignant hypertension were ruled out. Both the PRA and aldosterone levels did not exhibit significant suppression during the captopril challenge test (CCT; oral intake of 50 mg of captopril) and saline infusion test (SIT; infusion of 2 L of 0.9% saline between 8 A.M. and 12 A.M.). Based on these observations, we suspected that a JCT on the superior pole of the right kidney was the root cause of the hypertension. Subsequently, we conducted selective renal venous sampling to assess the level of direct renin secretion to confirm this suspicion. We performed selective angiography during the selective venous sampling, and the results indicated that the PRA level was 52.1 ng/mL/h in the right superior pole renal vein, 12.7 ng/mL/h in the right lower pole renal vein, 13.9 ng/mL/h in the left renal vein, and 11.5 ng/mL/h in the lower inferior vena cava (**Figures 1B, C**). These findings strongly suggested that the tumor in the superior pole of the right kidney directly released renin.

A laparoscopic tumor resection partial nephrectomy was carried out. The specimen weighed nearly 700 mg, and the tumor measured 5 × 6 × 8 mm in size. A pathological analysis revealed that the tumor cells originated from the juxtaglomerular region (**Figure 1D**). Immunohistochemical staining showed that the tumor cells were positive for CD34 and SMA.

Following the surgery, the PRA level returned to a normal value of 0.63 ng/mL/h, the potassium levels reached 3.9 mmol/L without supplementation, and the headaches experienced were relieved. On the first day after the operation, the blood pressure was 110/60 mmHg without the use of antihypertensive drugs. By the 7th postoperative day, the blood pressure was 105/60 mmHg, the potassium levels were at 5.2 mmol/L, and the PRA level was 0.74 ng/mL/h, at which point the patient was discharged. At follow-up almost 2 years after the nephrectomy, the creatinine level was normal, urine microalbumin was negative, and the blood pressure was 120–140/70–90 mmHg with nifedipine (30 mg/day).

## Discussion

Juxtaglomerular cell tumor originates from the smooth muscle cells of the afferent arteriole within the juxtaglomerular apparatus. These tumors are consistently benign neoplasms and secrete the enzyme renin, resulting in the hyperactivation of the renin–angiotensin–aldosterone system (3). Nevertheless, some non-renal malignant tumors, such as pancreatic cancer and ovarian cancer, can also secrete excessive renin (4, 5).

There have been fewer than 200 reported cases of JCT (2), and it exhibits a 2:1 preference for women, with a higher occurrence among adolescents and young adults. JCT can be categorized into three types, and they often manifest with both hypertension and hypokalemia. Only one atypical case has been identified where neither hypertension nor hypokalemia was present, but it was confirmed as nonfunctional JCT (6). Although resistant hypertension is the primary manifestation of JCT, the severity of hypertension varies among individuals and disease stages (7–9). Moreover, the degree of hypertension does not consistently correlate with the size of the tumor (10).

Both CT and MRI scans are useful to detect the presence of JCT (11). In this case, the tumor was not detected in a non-enhanced CT scan performed 20 years prior to the diagnosis. Two potential contributing factors make it challenging to detect JCT. Firstly, the small size of the tumor; the average diameter of JCT is reported to be 3 cm, whereas in this case, it was only 0.6 cm. Detecting tumors smaller than 0.5 cm in diameter on CT and/or MRI is a challenging task (12). Secondly, the tumor’s density appears isodense or hypodense in comparison to the renal medulla in non-enhanced CT scans. The dynamic enhanced CT and/or MRI scans differentiate JCT based on the contrast enhancement patterns in the imaging (13); however, the MRI has the characteristics of multi-directional and multi-sequence imaging, which has a higher resolution than CT (14).

Selective renal venous sampling helped locate the small JCT in this case and entirely ruled out other renal tumors seen on CT and MRI, such as angiomyolipoma or renal cell carcinoma (15, 16). In this particular case, the level of plasma renin activity in the right superior pole renal vein was 4.1- and 3.7-fold higher than that in the right lower pole renal vein and left renal vein as determined by selective renal venous sampling. A previous study showed that a lateralization ratio of 1.5 from renal venous sampling had a sensitivity of 56% and a specificity of 94% (17). These findings strongly support the use of selective renal venous sampling to identify and localize tumors as well as confirm direct renin secretion by the tumor in the superior pole of the right kidney. It is worth noting that the positive rate of renal venous sampling varies from 8.3% to 64%, depending on the localization (18–20).

In a healthy physiological state, renin secretion by juxtaglomerular cells is typically regulated by baroreceptors, the sympathetic nervous system, and the renin–angiotensin–aldosterone system. This regulation helps maintain blood pressure and electrolyte balance (21). However, in cases of JCT, renin secretion is autonomous, leading to resistant hypertension and hypokalemia in clinical settings. In this case, the levels of PRA and aldosterone were not significantly suppressed in CCT and SIT, suggesting that renin secretion by JCT is primarily autonomous, with some partial physiological regulation through angiotensin II mechanisms. It is worth noting that the mRNA expression of angiotensin II was not examined in this case (22).

The optimal treatment for JCT is surgical intervention, and laparoscopic resection or partial nephrectomy is often recommended due to the benign nature and size of the tumors (23, 24). In this particular case, the JCT was too small and entirely endophytic to be located *via* laparoscopy, but it was successfully identified through dynamic enhanced MRI. The pathological examination confirmed the presence of JCT, with positive CD34 and SMA markers, consistent with other reports (25, 26). After the tumor resection, there was a notable improvement in the patient’s resistant hypertension and hypokalemia. It is interesting to note that while most JCTs are benign, a small percentage can be malignant (27–30). About 10% of cases may still experience hypertension after surgery, which could be attributed to essential hypertension or other secondary factors, such as obstructive sleep apnea hypopnea syndrome or primary aldosteronism (17).

## Conclusion

In conclusion, we present a case of JCT that remained undiagnosed for nearly 20 years, likely due to the tumor’s small size and limitations in imaging techniques, and the diagnosis of the case was preoperatively identified by using MRI and confirmed with selective renal venous sampling. Resistant hypertension, especially when accompanied with hypokalemia, should prompt an investigation into its etiology. JCT is a rare and challenging disease to diagnose. Comprehensive examinations, including physical assessment, biomarkers, dynamic enhanced CT, and MRI, as well as selective renal venous sampling, are crucial for an accurate diagnosis.

## Data availability statement

The datasets presented in this study can be found in online repositories. The names of the repository/repositories and accession number(s) can be found in the article/supplementary material.

## Ethics statement

Ethical approval was not required for the studies involving humans because this was a rare case report, which did not require an ethical approval. The studies were conducted in accordance with the local legislation and institutional requirements. Written informed consent for participation was not required from the participants or the participants’ legal guardians/next of kin in accordance with the national legislation and institutional requirements because this was a rare case report, which did not require an ethical approval. Written informed consent was obtained from the individual(s) for the publication of any potentially identifiable images or data included in this article.

## Author contributions

D-EY: Writing – original draft. H-BH: Writing – review & editing. J-PG: Funding acquisition, Writing – review & editing. Y-LW: Writing – review & editing. D-PP: Writing – review & editing. H-HZ: Writing – review & editing. X-ZZ: Writing – review & editing. J-XF: Writing – review & editing. M-LW: Writing – review & editing. XL: Writing – review & editing. Y-FS: Writing – review & editing.
